# Comparison of Spectral CT and MRI in Pelvic Ring Fragility Fractures: A Prospective Diagnostic Accuracy Study

**DOI:** 10.3390/jcm13185446

**Published:** 2024-09-13

**Authors:** Mark Unthan, Bernhard W. Ullrich, Camilla Heinen, Felix C. Kohler, Philipp Schenk, Tobias Franiel, Florian Bürckenmeyer

**Affiliations:** 1 Department of Trauma, Hand and Reconstructive Surgery, Jena University Hospital, Friedrich Schiller University, Am Klinikum 1, 07747 Jena, Germany; 2 Department of Trauma and Reconstructive Surgery, BG Klinikum Bergmannstrost Halle gGmbH, Merseburger Str. 165, 06112 Halle, Germany; 3 Department of Research, BG Klinikum Bergmannstrost Halle gGmbH, Merseburger Str. 165, 06112 Halle, Germany; 4 Institute for Diagnostic and Interventional Radiology, Jena University Hospital, Friedrich Schiller University, Am Klinikum 1, 07747 Jena, Germany

**Keywords:** pelvic ring fragility fracture, spectral CT, dual-energy CT

## Abstract

**Background/Objectives:** Fragility fractures of the pelvis (FFP) are characterized by inadequate trauma to a structurally compromised bone, primarily in osteoporosis. Conventional CT studies can be inadequate in identifying FFPs. An MRI of the pelvis is considered the gold standard in diagnosing FFPs. Spectral CT or Dual-Energy CT may have comparable diagnostic accuracy. It provides additional insights into associated bone marrow edema. The aim of this prospective monocentric study is to evaluate the diagnostic accuracy of Spectral CT compared to the gold standard MRI in diagnosing FFP. **Methods:** Over a 2-year period, patients presenting in the emergency department with clinical suspicion of an FFP were consecutively included. They underwent Spectral CT (GE Revolution 16 cm GSI) upon admission, followed by an MRI. The gold standard for diagnosing FFP is pelvic MRI, showing sensitivity and specificity ranging from 97% to 100%. The acquired images were evaluated and classified using the osteoporotic fractures of the pelvis (OFP) classification. **Results:** Compared to the reference test, which was the MRI pelvis, the sensitivity of the CT pelvis was determined to be 86.8 (95% confidence interval (CI) 71.9–95.6%) with a specificity of 84.6% (95% CI: 54.6–98.1%, *p* = 0.453). Spectral CT could identify an additional FFP correctly, exhibiting a sensitivity of 89.5% (95% CI: 75.2–97.1%, *p* = 0.688), while maintaining the same specificity as the conventional CT. The inter-rater reliability assessment for Spectral CT, conducted by four independent raters, resulted in a Fleiss’ Kappa value of 0.516 (95% CI: 0.450–0.582, *p* < 0.001). **Conclusion:** The sensitivity of Spectral CT in the detection of pelvic ring fragility fractures shows a slightly lower sensitivity compared to MRI. There were no statistically significant differences observed when compared to conventional CT or MRI. In conclusion, Spectral CT may be beneficial in distinguishing FFP, particularly in cases where a definitive diagnosis is uncertain. Level of Evidence: II.

## 1. Introduction

Demographic changes in most industrialized countries have led to an increase in pelvic ring fragility fractures [1,2]. Fragility fractures are characterized by low-energy trauma in a structurally compromised bone, primarily associated with osteoporosis [3]. Pelvic ring disruptions are predominantly observed in young working patients due to high-energy blunt trauma [1,4]. Conversely, fragility fractures typically occur after low-energy trauma, such as a ground-level fall, and generally do not cause damage to soft tissues, intrapelvic organs, or life-threatening hemorrhage [1,5]. However, pelvic ring fragility fractures pose a significant challenge in a highly demanding population, characterized by decreased physiological reserve and increased frailty, often clinically presenting with multimorbidity and polypharmacy [6].

Impaired bone density in the elderly population, particularly in the Os sacrum, leads to the so-called alar voids [7,8]. They present in the paraforaminal lateral region of the sacral alar from S1 to S3 with significantly decreased bone density. Fragility fractures of the pelvis often involve the alar voids, mainly due to lateral compression mechanisms [1,4]. Besides the common AO Tile classification [9], there are two commonly used classifications for osteoporotic sacral and pelvic ring fractures like the fragility fractures of the pelvis (FFP) classification by Rommens [5] and the osteoporotic fractures of the pelvis (OFP) classification [10]. OFP provides substantial to perfect inter-rater and intra-rater reliability.

**Figure 1:**
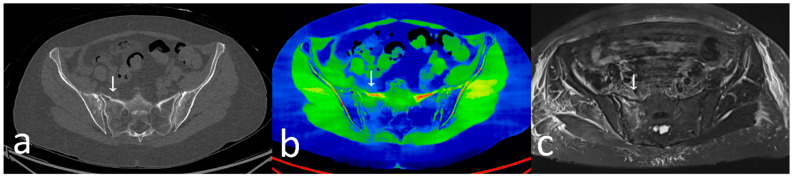
Image date from a patient with OFP III fracture, pictured in (**a**) a conventional CT slice, (**b**) Spectral CT with highlighted green bone marrow edema, and (**c**) MRI STIR sequence. Fractures are highlighted by an ↓.

Accurate diagnostic algorithms are crucial for effectively assessing the injury and determining appropriate nonoperative or operative treatment strategies [11]. Plain X-ray films identify sacral fragility fractures in only 20–38% of cases [12]. Conventional CT studies may also fall short in identifying fractures of the posterior pelvic ring, with sensitivity ranging from 60 to 75% [12,13,14,15,16]. MRI of the pelvis is considered the gold standard in diagnosing fractures of the posterior pelvic ring, demonstrating a sensitivity of nearly 100% [12,13]. Spectral CT or Dual-Energy CT, a novel diagnostic tool in musculoskeletal imaging, provides additional insights into associated bone marrow edema and is believed to offer comparable diagnostic accuracy in detecting pelvic ring fragility fractures to MRI (Figure 1) [13,14,15,16,17].

Spectral CT combines the principles of conventional CT and the differentiation of tissue based on their different X-ray absorption properties at two energy levels. The radiation dosages of a Spectral CT are within standard references for CT scans [18].

The aim of this prospective monocentric study is to evaluate the diagnostic accuracy of Spectral CT of the pelvis compared to the gold standard MRI of the pelvis in diagnosing pelvic ring fragility fractures.

## 2. Materials and Methods

### 2.1. Study Design and Participants

The patients were recruited in a consecutive series between April 2021 and February 2022 with a suspected fragility fracture of the pelvis in a prospective way in one study center. All patients underwent Spectral CT and MRI of the pelvis. Exclusion criteria were no known osteopenia or osteoporosis or normal Hounsfield units (>130 HU) in L5 as an indicator of reduced bone density in the CT scan [19,20,21], fracture due to high-energy trauma, known pre-existing bone marrow disease, or contraindication for MRI (pacemakers or claustrophobia).

All patients gave written consent.

### 2.2. Imaging Methods

The images were generated on a 256-slice, single-source, dual-energy CT scanner (GE Revolution 16 cm, GE HealthCare, Waukesha WI, USA) with rapid kilovolt switching [17]. The image reconstructions performed with GSI Xtream included a water map with hydroxylapatite subtraction, which were visualized in both axial and coronal views. To enhance the visualization of bone marrow edema, the maps were color-coded using the post-processing software GE AW-Server 3.2 Ext. 4.8. The rapid kilovolt switching technology allows the scanner to acquire images at different energy levels, specifically at 70 KeV and 110 KeV. This advanced technique enables rapid alteration and simultaneous acquisition of images, resulting in a significant reduction in radiation exposure [18]. Importantly, the radiation doses associated with Spectral CTs are comparable to those of single-energy CT scans.

Three different MRI scanners were used for diagnostics in our clinic during the study period: Siemens Magnetom Sola, Siemens Magnetom Vida, and Siemens Magnetom Aera (Siemens Healthineers AG, Erlangen, Germany). The extent of edema was examined with fluid-sensitive fat-saturated sequences (STIR/TIRM, PDw fatsat) axial and coronal and with additional T1w sequences axial and coronal.

The reference imaging for pelvis injury was chosen to be the MRI pelvis, known as the gold standard in diagnostics of fragility fractures of the pelvis [11,13,16,22].

The acquired images of conventional CT (70 KeV data), conventional CT with Spectral images, and MRI of the pelvis were pseudonymized and without clinical information graded in a randomized order by three orthopedic surgeons. The surgical team consisted of a 6-year postgraduate assistant, a 9-year postgraduate consultant, and an attending surgeon with over 20 years of experience in pelvic surgery. Additionally, an attending radiologist specializing in musculoskeletal imaging analyzed the data. Results were documented on a finding sheet for each image series.

In all three series, OF Pelvis classification was determined. The OFP classification was preferred over the FFP classification because it uniquely incorporates MRI and Spectral CT features for fracture classification, distinguishing it from the FFP classification by Rommens and the AO classification. Edema identified via Spectral CT or MRI can be classified as bone edema and included as a classifier [10]. OF pelvis type I injuries consist of pelvic ring edema, OF pelvis type II are isolated anterior pelvic ring injuries, OF pelvis type III are unilateral sacral fractures with or without anterior ring lesion, OF pelvis type IV are bilateral sacral fractures with or without anterior ring lesions, and OF pelvis type V fractures are iliac or sacroiliac fractures with or without anterior ring lesion. The reference was the results from the musculoskeletal radiologist.

### 2.3. Analysis

Data were collected in Microsoft Excel sheets (Microsoft Excel 365, Microsoft Corporation) and exported to SPSS (IBM SPSS Statistics for Windows, Version 29.0, IBM Corp, Armonk, NY, USA) for statistical analysis. The diagnostic accuracy of CT and Spectral CT were calculated by comparing results with those of MRI as the reference test, using cross tables for the results conducted by the musculoskeletal radiologist. The absence of a fracture in the posterior pelvic ring (OF Pelvis 0–2) was compared to the presence of a fracture in the posterior pelvic ring (OF Pelvis 3–5). The reference test for identifying fractures in the dorsal pelvic ring was the MRI pelvis. Based on the reference test, an observed fracture in the dorsal pelvic ring in either the conventional CT-Dataset or the Spectral CT-Dataset was labeled as a true positive case. A fracture observed only in the conventional CT or the Spectral CT and not in the MRI pelvis was labeled as a false positive. If neither modality showed a fracture, it was labeled as a true negative. False negatives were defined as a present fracture in the MRI pelvis with absence in the CT or Spectral CT. The statistical analysis was carried out using the McNemar Test.

The number of participants was determined due to a thorough literature review and the clinical capacities to evaluate and include the patients in a consecutive series [14,16].

The inter-rater reliability was calculated using Fleiss’ Kappa [23]. Unless otherwise stated, mean values ± standard deviation are used for descriptive purposes. The minima and maxima are given as a range to show the validity range of our data. The values for reliability, sensitivity, and specificity are given as the mean and its 95% confidence interval (lower and upper limit).

## 3. Results

**Figure 2:**
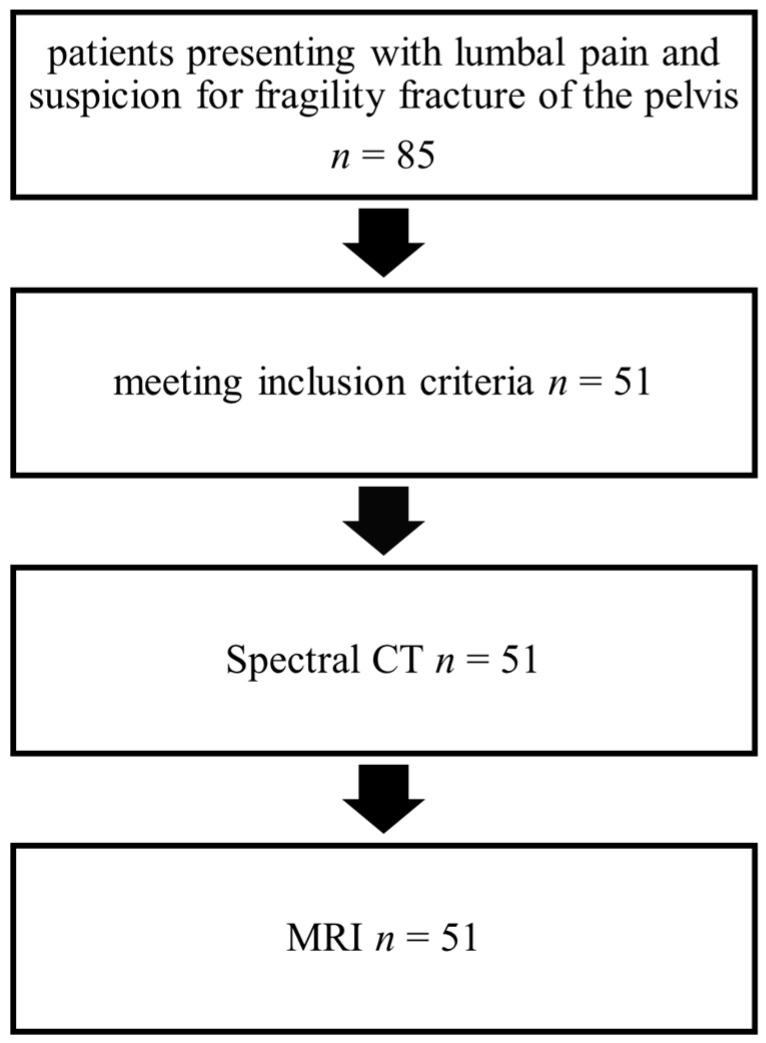
The flow of patients to investigate the diagnostic accuracy of the Spectral CT compared to MRI.

Of 85 patients, 51 met the inclusion criteria. Fifty-one patients were enrolled in the study comprising 35 women and 16 men. The average age at the time of presentation was 80 ± 10 years (range: 54 years to 94 years). All participants underwent a Spectral CT upon admission, followed by an MRI scan of the pelvis after an average period of 2 ± 3 days (range: 0 to 13 days) (Figure 2). The measurement of the L5 vertebrae yielded an average of 68 ± 30 Hounsfield units (range: 10 HU to 110 HU).

In this study, eight patients had no fragility fracture of the pelvis. Their pain was attributed to various causes, such as osteoporotic fractures of the lumbar spine. Among the 43/51 (84%) a fracture or edema in the pelvic ring was observed (Table 1). Eight patients were included while not showing a pelvic ring fracture. Four (8%) out of fifty-one patients presented with an isolated fracture of the anterior pelvic ring, either at one side or bilateral (OFP 2). One patient exhibited only edema in the posterior pelvic ring (OFP1). Alterations in the posterior pelvic ring were detected in 36 out of 51 (71%) patients in this study population, and a bilateral sacral fracture was identified in 11 out of 51 of the cases (22%).

### 3.1. Diagnostic Accuracy

Compared to the reference test (the MRI pelvis), the sensitivity of the CT pelvis was determined to be 87% (95% CI: 72–96%) with a specificity of 85% (95% CI: 55–98%, *p* = 0.450) using the McNemar Test while comparing the presence of an OF pelvis 0–2 and an OF pelvis 3–5 injury as shown in Table 2.

Spectral CT could identify an additional fracture in the dorsal pelvic ring correctly, exhibiting a sensitivity of 90% (95% CI: 75–97%, *p* = 0.683) while maintaining the same specificity as the conventional CT (Table 3).

### 3.2. Inter-Rater Reliability

The inter-rater reliability assessment for Spectral CT, conducted by four independent raters, resulted in a Fleiss’ Kappa value of 0.516 (95% CI: 0.450–0.582, *p* < 0.001), indicating a moderate level agreement among the raters [23]. As presented in Table 4, the sensitivity in detecting a dorsal pelvic ring fracture varied from 69% to 97%.

## 4. Discussion

The aim of our prospective clinical study was to evaluate the diagnostic accuracy of the Spectral CT to MRI of the pelvis. The Spectral CT was hypothesized to offer advantages in detecting posterior pelvic ring fractures over conventional CT [15,16].

We were able to see that Spectral CT had a sensitivity of 90%, which was slightly higher than the sensitivity of 87% observed with conventional CT. This resulted in the correct identification of one additional fracture in the posterior pelvic ring within our study population. In our investigation, we demonstrated that Spectral CT exhibited a sensitivity of 90%, marginally surpassing the 87% sensitivity of conventional CT. As far as we are aware, there are currently no prospective studies addressing this matter. In a retrospective examination, Palm et al. reported an almost 100% sensitivity for Spectral CT [16]. Our analysis not only reaffirms the diagnostic efficacy of Spectral CT with a dependable sensitivity but also sheds further light on its true clinical significance through modifications in our study design.

Spectral CT may encounter challenges with noisy images and out-of-field artifacts in obese patients, particularly when the patient diameter exceeds 50 cm, potentially leading to difficulties in accurate diagnosis. Parakh et al. also highlighted that images are considerably noisier in these cases. Stronger beam hardening artifacts and out-of-field artifacts as well as photon starvation especially in the water maps likely contribute to the difficulty in achieving correct diagnoses during scans of obese patients with a patient diameter exceeding 50 cm [24,25]. It is known that the ability of conventional CT to detect fractures posterior pelvic ring is limited. Previous studies have reported lower sensitivities for conventional CT, ranging from 53% [13] up to 75% [11,12,13,14,15,16]. Most of these studies were retrospective analyses comparing CT with MRI. The recommendation for MRI in pelvic ring fragility fractures results from inconclusive cases with the need for clarification of a fracture in the posterior pelvic ring. In our prospective study, we encountered minimal cases with clinical uncertainty, contributing to the relatively high sensitivity of conventional CT at 87%. This finding suggests that the need for additional imaging, such as MRI, may be less urgent than previously assumed.

In our clinical experience, a diagnostic test for pelvic ring fractures with a sensitivity of nearly 90% is considered very acceptable. The added clinical advantage in a routine MRI pelvis with every fragility fracture is debatable. The sensitivity of the CT and Spectral CT scan could be enhanced by considering clinical information like pain and/or weight-bearing ability [26].

We utilized the GE Revolution CT with the rapid kilovolt switching technique to acquire and process our images. Other technical approaches are available to acquire Spectral images like the two-tube and two-detector technique as employed by Siemens or the multilayer detector technique by Philipps Medical CT may result in different imaging capabilities [15].

Spectral images were found to aid the diagnosis of pelvic ring fragility fractures among healthcare professionals [15,16]. Our study demonstrated moderate inter-rater reliability, with sensitivities ranging from 70% to 97% among all raters. Although all raters were familiar with the modality, some differences remained.

The evaluators reviewed conventional 2D images for their assessments. However, it is worth noting that further processing and imaging in high-resolution 3D scans may simplify report generation and enhance the recognition of complex fracture patterns. The study is confined to a relatively small number of included patients. However, to the best of our knowledge, there is no larger prospective cohort of selected patients. Increasing the sample size may unveil statistically significant differences among test results. A well-designed multicenter study could increase the sample size significantly. Various examinations were not conducted by the same study team. We established a standard operating procedure for Spectral CT, including using a pelvic binder for obese patients; however, the quality of Spectral images varies based on patient positioning. Our experienced radiologist performed post-processing to emphasize bone marrow edema and reduce artifacts. The study predominantly comprised women; therefore, increasing male participant representation would be beneficial.

It is important to consider that fragility fractures represent a challenging clinical issue that requires decisive diagnostics and various therapeutic approaches. Based on our results, there is no clear evidence supporting routine MRI scans following a fragility fracture if there is supporting clinical and radiographic data. Spectral CT may be beneficial in cases with clinical uncertainty. In our clinic, Spectral CT is routinely acquired for fragility fractures to obtain additional information and minimize the need for additional MRI in uncertain cases. Many patients with contraindications for MRI or an increased risk of complications associated with MRI, such as those with pacemakers set to pause, may benefit from the Supplementary Spectral images. However, experienced clinicians and radiologists are necessary to accurately pinpoint and interpret clear pathologies.

Further clinical utilization and improved technical solutions for acquiring and processing Spectral data are required to further evaluate the potential of this promising technique.

## 5. Conclusions

The sensitivity of Spectral CT in the detection of pelvic ring fragility fractures shows a slightly lower sensitivity compared to MRI. There were no statistically significant differences observed when compared to conventional CT or MRI. The inter-rater reliability of Spectral CT showed a moderate level of agreement. In conclusion, Spectral CT may be beneficial in distinguishing pelvic ring fragility fractures, particularly in cases where a definitive diagnosis is uncertain.

## Figures and Tables

**Table 1:** OF pelvis classification in the study population.

OFP Classification (Based on MRI)	*n*
0	8
1	1
2	4
3	26
4	11
5	1

**Table 2:** Diagnostic accuracy of the conventional CT pelvis (index test) compared to MRI pelvis (reference test).

		MRI Pelvis		
		OF Pelvis 0–2	OF Pelvis 3–5	*n*
Conventional CT	OF Pelvis 0–2	11	5	16
	OF Pelvis 3–5	2	33	35
	*n*	13	38	51

**Table 3:** Diagnostic accuracy of the Spectral CT pelvis (index test) compared to MRI pelvis (reference test).

		MRI Pelvis		
		OF Pelvis 0–2	OF Pelvis 3–5	*n*
Spectral CT	OF Pelvis 0–2	11	4	15
	OF Pelvis 3–5	2	34	36
	*n*	13	38	51

**Table 4:** Diagnostic accuracy of the Spectral CT by different raters.

	Rater 1	Rater 2	Rater 3	Rater 4
	Radiologist	Orthopedic Surgeon	Orthopedic Surgeon	Orthopedic Surgeon
Sensitivity	89% (75–97%)	97% (86–99%)	85% (65–96%)	69% (52–84%)
Specificity	85% (55–98%)	86% (57–98%)	48% (28–69%)	87% (60–98%)
Positive Predictive Value	94% (82–98%)	95% (83–98%)	63% (53–72%)	93% (77–98%)
Negative Predictive Value	73% (51–87%)	92% (63–99%)	75% (53–89%)	54% (41–67%)

## Data Availability

The original contributions presented in the study are included in the article. Further inquiries can be directed to the corresponding author.
